# Chronological occurrence of PI3KCA mutations in breast cancer liver metastases after repeat partial liver resection

**DOI:** 10.1186/s12885-019-5365-2

**Published:** 2019-02-22

**Authors:** Aldrick Ruiz, Mylène Sebagh, Raphaël Saffroy, Marc-Antoine Allard, Nelly Bosselut, Giulia Hardoin, Julie Vasseur, Jocelyne Hamelin, René Adam, Jean-François Morère, Antoinette Lemoine

**Affiliations:** 1 Department of Surgery, University Medical Center Utrecht, Utrecht, The Netherlands; 2 AP-HP Hôpital Paul Brousse, Centre Hépato-Biliaire, Villejuif, France; 3 Department de Pathologie, AP-HP Hôpital Paul Brousse, Villejuif, France; 4 Inserm UMR-S 1193, Université Paris-Sud, Orsay, France; 5 AP-HP Hôpital Paul Brousse, Department Oncogénétique, Villejuif, France; 6 Inserm UMR-S 985, Université Paris-Sud, Orsay, France; 7 Department. Cancérologie, AP-HP Hôpital Paul Brousse, Villejuif, France; 8 Departement of Oncogenetics, APHP, GH Paris-Sud, Hôpital Paul Brousse, Inserm UMR-S 1193, Université Paris-Saclay, 14 Avenue Paul Vaillant Couturier, 94800 Villejuif, France

**Keywords:** PI3KCA, Breast, Cancer, Liver, Metastases, Lineage, Evolution

## Abstract

**Background:**

Liver metastases of breast cancer are frequent and can recur even after “complete/R0” resection in combination with systemic and hormonal treatments. The aim of this study was to analyze throughout repeat hepatectomies for liver metastases the evolution of PI3KCA gene mutational status.

**Methods:**

All liver metastases nodules (*n* = 70) from 19 women who underwent at least 2 liver resections were reexamined. DNA extraction from archived tumoral tissue was performed and the major ‘hot spot’ mutations in the helical and catalytic domains of PI3KCA have been analyzed using Massarray platform (Agena Bioscience) based on allelic discrimination PCR amplification followed by sensitive mass spectrometry detection.

**Results:**

The two major somatic hot spot *PI3KCA* mutations were found in 27 (38.6%) nodules corresponding to 8 of the 19 patients (42%). The frequency of women whose breast cancer liver metastases (BCLM) carries PI3KCA mutations increased from the first to the third hepatectomy. Tumors carrying PI3KCA mutations are significantly larger and more frequently observed when resections were R0 compared to patients with no PI3KCA mutation.

**Conclusion:**

PI3KCA mutations are frequently observed in BCLM and persist along with the recurrence. Their identification in circulating tumor cells should become a useful biomarker in the routine practice of breast cancer management to prevent tumor recurrence and overcome the problems of intra- and inter-tumoral heterogeneity of the current biomarkers,

## Introduction

A significant proportion of breast cancer patients will eventually develop metastases (stage IV) with poor prognostic outcome [1–3]. Distant metastatic sites were identified as the brain, liver, lungs, and bones. Once metastatic disease is diagnosed, treatment is generally palliative and usually consists of anthracycline- or taxane-based regimes with or without hormonal or targeted therapeutic agents. This widely implemented palliative approach has yielded median survival ranging between 3 to 16 months [2–7]. For liver metastases, aggressive approaches adopted by experienced hepatobiliary centers, where systemic and hormonal treatments for metastatic breast cancer are combined with surgical removal, has shown promising results in improving patient survival [8–12]. However, of the patients who undergo removal of breast cancer liver metastases, a portion still develops tumor recurrence in the liver.

Genomic profiling of tumor tissues has been increasingly used to understand and investigate the evolution of metastatic disease and alternative targets for breast cancer therapies [13, 14]. There are several studies describing genotype profiling of breast tumors. Frequently altered genes are ErbB2, PI3K (phosphatidylinositol 3 kinase) pathways, TP53, BRCA1/2, and PTEN [15]. However, following common clinical practice there has few studies examining tumors beyond the usual point of first metastatic presentation or in recurrences after removal [16–19]. PIK3CA mutations have been reported to be present in over one-third of cases, with enrichment in the luminal and in human HER2-positive subtypes [19]. In human tumors or mammary epithelial cell lines, the two most common mutant alleles (H1047R and E545K) were found to activate PI3K signaling and to be involved in tumorigenesis and resistance to chemotherapy [20–23]. Therefore, the aim of this study was to analyze the chronology of major hotspot mutations in PI3KCA occurrence in a series of patients who underwent at least 2 liver resections for breast cancer liver metastases.

## Material and methods

As part of treatment patients with recurrent breast cancer liver metastases can undergo repeat liver resection. This study is a retrospective case series experiment.

### Case selection

**Fig. 1:**
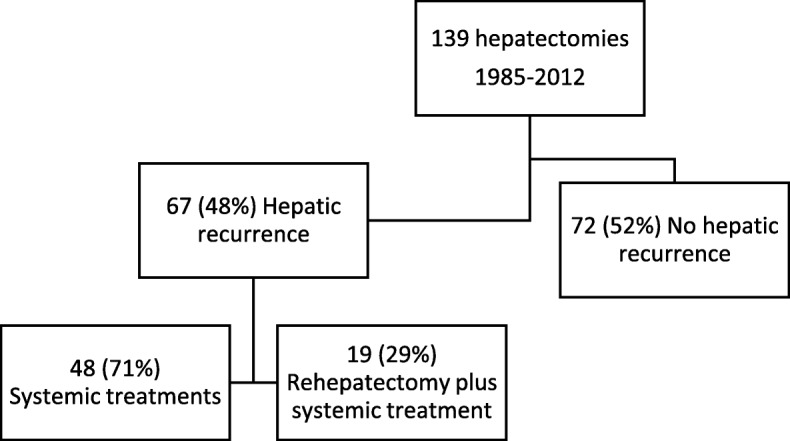
Flowchart of the study population

Study size was based on convenient sample of the experience of a hepatobiliary center. All consecutive patients with breast cancer liver metastases (BCLM) who underwent at least two separate liver resections at our center between January 1985 and December 2012 were included in the study (Fig. 1). Patients were selected from our prospectively maintained institutional database, and each medical record was reviewed to update basic clinical and pathological data.

### Tissue samples

Representative tumor samples of all nodules within each patient were collected and reviewed to examine genetic abnormalities. Hematoxylin-eosin-stained slides from tumors were assessed for the ratio percentage of tumor cells/sample area (non-tumor tissue, stroma of the tumor) by pathologists at our department. Three sections of 10 μm thickness were obtained from the paraffin-embedded tissue containing at least 50% of tumor cells. Vascular or lymphatic invasion features were also analyzed. Immunohistochemistry analysis for hormonal receptors was conducted to analyze expression of hormonal receptors and HER2.

### DNA extraction

DNA extraction was performed using the QIAmp DNA Mini kit (Qiagen, France), which provides silica-membrane-based nucleic acid purification [20].

### Mutations detection

PIK3CA and HER2 mutations was analysed using a Massarray iPlex technology panel and Massarray online design tools (Agena Bioscience). The panel includes main exons (9 and 20) PIK3CA mutations (all mutations from 542 to 545–546-1047 codon), exon 20 HER2 insertions (codons 774–776) and exon 2 to 4 KRAS. The Massarray iPlex procedure involves a three-step process consisting of the initial PCR reaction, inactivation of unincorporated nucleotides by shrimp alkaline phosphatase and a single-base primer extension. Then, the products are nano-dispensed onto a matrix-loaded silicon chip (SpectroChipII, Ageno Bioscience) and finally, the mutations are detected by MALDI–TOF (matrix-assisted laser desorption-ionization–time of flight) mass spectrometry. Data analysis was performed using MassArrayTyper Analyzer software 4.0.4.20 (Agena Bioscience, Hamburg, Germany), which facilitates visualization of data patterns as well as the raw spectra. To allow detection of rare PIK3CA, HER2 or KRAS mutations, analysis of the whole exons was completed by High Resolution Melting analysis (HRM). PCR was performed in a 96-well plate with a 20 μL volume including 50 ng DNA, 2 mmol/L of each primer, 2.5 mmol/L of MgCl2, 4.7 μL of water, and 10 μL of LightCycler 480 HRM Master mix (Roche Diagnostics, France). The reaction mix was subjected to initial denaturation at 95 °C for 10 min, followed by 45 cycles of amplification consisting of denaturation at 95 °C for 30 s, annealing at 58 °C for 30 s, and extension at 72 °C for 30 s. Melting was performed with a denaturation step at 95 °C for 1 min, followed by annealing at 40 °C for 1 min and a melt from 70 °C to 97 °C at a ramp rate of 0.03 °C/second with 18 acquisitions per second. LightCycler 480 Resolight Dye (Roche), a fluorescent dye that uniformly binds to the minor groove of double-stranded DNA in a nonsequence-dependent manner for melting analysis was used. In all experiments, positive and negative controls were included (Horizon diagnostics).

### Clinical data

Hepatectomies were conducted in a tertiary center where data were available, extra information regarding the primary tumor was collected. Chronology of primary tumor, first liver metastasis, first hepatic resection, hepatic recurrences and repeat hepatectomies for each case were used for interval calculation.

### Statistical methods

Categorical variables were compared between groups by the chi-square test or Fisher’s exact test when appropriate and continuous variables were compared using the independent-sample t-test. All statistical analyses were performed with SPSS version 21.0 (SPSS Inc., Chicago, IL, USA).

## Results

### Patient samples

Between 1985 and 2012, 139 female patients underwent partial liver resection for breast cancer liver metastases at our institution (Fig. 1). Sixty-seven women (48%) developed hepatic recurrence of which 19 patients (28%) had subsequent second liver resections with curative intent. Four of them had a third hepatectomy. A total of 86 breast cancer liver metastases nodules were removed. Sixteen nodules were not available for molecular analysis due to insufficient tumoral cell content or inability to amplify DNA. High Resolution Melting and allelic discrimination PCR amplification using Massarray analyses were performed on samples originating from 70 nodules.

### Mutations discovered

**Fig. 2:**
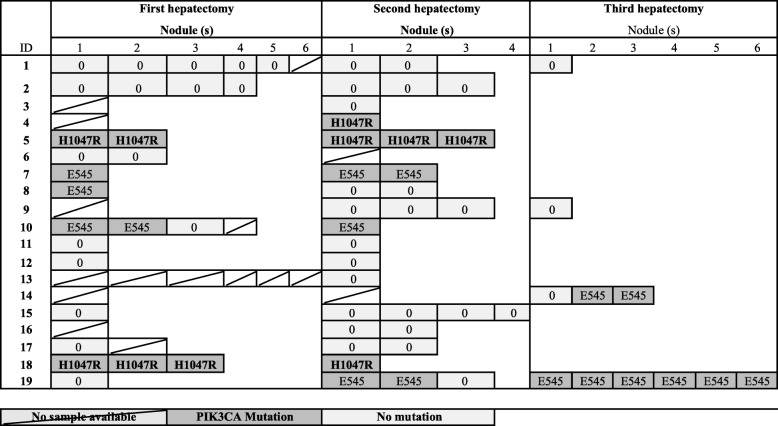
Mutation detected in corresponding nodule after hepatectomies

Overall, the two major somatic hot spot mutations in the helical and catalytic domains of *PI3KCA* in the breast cancer liver metastases were found in 27 (38.6%) nodules corresponding to 8 of the 19 patients (42%) (Fig. 2). No mutation in HER2 (exon 20) or KRAS (exon 2 to 4) were detected both by the High Resolution Melting analysis and allelic discrimination.

### Population characteristics

Table 1 summarizes clinical characteristics of the patient population stratified by their corresponding hepatectomy. Mean maximum size of tumors removed during first hepatectomy was significantly larger in patients with PI3KCA mutation compared to patients with no PI3KCA mutation 33 mm vs. 17 mm respectively *p* = 0.07. At the Second hepatectomy the proportion of patients treated with anti-HER2 targeted therapy is significantly higher in patients without PI3KCA compared to patients with PI3KCA mutation 82% vs. 17% respectively (*p* = 0.035). Timeline, anatomical location and extend of metastatic disease are highlighted for the presence of mutation and is presented in Table 2.

**Table 1:** Clinical characteristic according to PI3KCA mutation status

NN	First hepatectomy				Second hepatectomy					Third hepatectomy				
	No Mutation		Mutation		No Mutation			Mutation		No Mutation			Mutation	
	*N* = 8		*N* = 5		*N* = 11			*N* = 6		*N* = 2			*N* = 2	
Hepatic metastases														
Mean age at resection + SD Years	42 + 7		44 + 7			51 + 11		50 + 12			63 + 18		42 + 4	
Mean interval between primary removal and liver resection + SD Months	42 + 20		47 + 37			43 + 21		46 + 33			28 + 9		22 + 9	
Mean number of BCLM diagnosed + SD Distribution	2 + 2		2 + 2			2 + 2		2 + 1						
Unilateral	5	50%	4	80%		9	82%	4	67%		1	50%	1	100%
Bilateral	5	50%	1	20%		2	18%	2	33%		1	50%	0	0%
Concomitant extra-hepatic disease														
No	10	91%	4	80%		8	73%	4	67%		1	50%	1	100%
Yes	1	9%	1	20%		3	27%	2	33%		1	50%	0	0%
Chemotherapy before liver resection														
No	2	18%	1	20%		0	0%	1	17%		0	0%	0	0%
Yes	9	82%	4	80%		11	100%	5	83%		2	100%	2	100%
Hormonal Therapy before liver resection														
No	8	73%	2	40%		2	18%	2	33%		1	50%	0	0%
Yes	3	27%	3	60%		9	82%	4	67%		1	50%	2	100%
Targeted Therapy before liver resection										**				
No	7	64%	4	80%		2	18%	5	83%		1	50%	2	100%
Yes	4	36%	1	20%		9	82%	1	17%		1	50%	0	0%
Histopathological characteristics														
Mean maximum resected tumor size + SD, mm	17 + 15		33 + 15		*	21 + 16		26 + 12			19 + 8		23 + 11	
Resection margin***														
R0	8	89%	3	60%		8	80%	4	67%		0	0%	2	100%
R1	1	11%	2	40%		2	20%	2	33%		1	100%	0	0%
Microscopic intrahepatic invasion														
No	6	60%	1	20%		7	64%	2	33%		–		0	0%
Yes	4	40%	4	80%		4	36%	4	67%		–		2	
Cell surface receptors status														
Estrogen														
Negative	0	0%	2	40%		4	36%	2	33%		0	0%	1	50%
Positive	8	100%	3	60%		7	64%	4	67%		2	100%	1	50%
Progesterone														
Negative	5	63%	4	80%		6	55%	2	33%		1	50%	1	50%
Positive	3	38%	1	20%		5	45%	4	67%		1	50%	1	50%
Her2/Neu														
Negative	6	75%	4	80%		6	60%	4	67%		0	0%	2	100%
Positive	2	25%	1	20%		4	40%	2	33%		1	100%	0	0%
Differentiation														
Well differentiated	0	0%	1	20%		1	14%	2	40%		1	50%	0	0%
Moderately differentiated	5	71%	4	80%		3	43%	2	40%		0	0%	1	50%
Poorly differentiated	2	29%	0	0%		3	43%	1	20%		1	50%	1	50%

* *P* < 0.1 ** *P* < 0.05 *** R0: complete surgical resection with a negative surgical margin at histopathology; R1: invaded surgical margins

**Table 2:** Clinical characteristics and presence of PI3KCA mutations

ID	Year Primary tumor		First hepatectomy				Second hepatectomy				Third hepatectomy			Survival since primary (months)
		Margins	Interval (months)^a^	Intra hepatic Location	xhep	Margins	Interval (months)^a^	Intra hepatic Location	xhep	Margins	Interval (months)^a^	Intra hepatic Location	xhep	
1	1989	R0	45	BI	No	–	53	BI	No	–	21	UL	No	140
2	1998	R1	65	UR	Yes	R0	40	UR	No					142
3	2002	–	22	UR	No	R0	57	UR	No					95
4	1984	–	65		No	R0	46	BI	No					195
5	1988	R0	27	UL	No	R1	26	UL	No					127
6	1998	R0	49	UR	No	R0	38	BI	Yes					112
7	1992	R0	46	UR	Yes	R0	28	UL	No					108
8	2005	R1	34	UR	No	R0	34	UR	Yes					93
9	1988	R0	30	UR	No	R1	34	UL	No	R1	34	BI	Yes	205
10	1993	R0	56	UL	No	R1	113	UL	Yes					182
11	2000	R0	34	UL	No	R1	36	UL	No					107
12	1998	R0	27	BI	No	R0	49	UL	No					120
13	1999	–	17	BI	No	R0	29	BI	Yes					72
14	1986	R0	82	UL	No	R0	40	UL	No	R0	28	UR	Yes	244
15	1999	–	62		No	R0	92	UL	Yes					157
16	1997	R0	60	UR	No	R0	44	UR	No					145
17	1992	R0	42	BI	No	R0	8	UR	No					75
18	2003	R1	10	BI	No	R0	32	BI	Yes					63
19	1990	R0	36	BI	No	R0	32	UR	No	R0	15	UR	No	93

^a^To previous event, xhep extra hepatic metastases, BI Bilobular, UL Unilateral Left, UR Unilateral Right, PI3KCA mutated tumors

### Chronological occurence of PI3KCA mutation

Four out of 5 patients with “Breast Liver Cancer Metastases” with *PI3KCA* mutations at the first hepatectomy, had the same mutation at the second hepatectomy. Three (16%) women had de novo *PI3KCA* in their tumors at the second or third hepatectomy. Four patients received a third hepatectomy of which 2 had *PI3KCA* mutations (one possible de novo and one recurrence). At the first, second and third hepatectomy, 38, 47 and 50% respectively of the patients for whom tumors were available had *PI3KCA* mutations (Fig. 2).

Of the 8 multinodular hepatectomies with *PI3KCA* mutation, the same mutation was found in all the nodules for 5 of them (62.5%). For the 3 remaining hepatectomies, the *PI3KCA* mutation was observed in 2 out of 3 nodules.

## Discussion

In our case series of patients who underwent repeat hepatectomies for breast cancer liver metastases, PIK3CA hotspot mutations were frequently observed in most of the nodules for multinodular tumors and persist along with the liver metastases recurrence. Their frequencies also increased along with hepatectomies. When examining the specific mutation H1047R or E545K, mutation remains the same within each patient over time.

The global frequency of PI3KCA mutations found in liver metastases in this series of patients is similar to those reported in sequencing studies in multiple cancers. PIK3CA mutations are present in approximately 30% of all breast cancers, with a higher frequency reported in oestrogene or progesterone positive tumors as well as in HER2–positive breast cancers [13–19]. In our series, almost all tumors express either HER2 or a hormone receptor, explaining the high frequency of PI3KCA gene mutations.To our knowledge, there are no data reporting neither frequencies of PI3KCA mutations in breast cancer liver metastases nor a specific gene signature including PI3KCA mutations. In the molecular classification of Hennecke et al., liver metastasis was most frequent in HER-2-enriched variety and PI3KCA mutations have also been enriched in HER-2 breast cancers [4]. We can observe a tendency for more PI3KCA mutated nodules between the first and second hepatectomies for tumors expressing these receptors.

The increase in the mutation rate along with hepatectomies can be considered as a marker of clonal evolution in favor of the selection of a dominant clone. In our study, mutations of PI3KCA show such an increase after each hepatectomy and especially a generalization within nodules for multinodular tumors. This increase may be due to malfunctions of the mechanisms for maintaining the integrity of the genome or its exposure to mutagenic agents and thus sign the resistance to treatments. We observe the expansion of mutations within nodules and at each hepatectomy highlighting a potential role of PI3KCA mutations as a marker of treatment resistance and expansion of clones bearing the PI3KCA anomaly. However, since the increase in the frequency of mutations is inversely correlated with the HER2 status of primary tumors that guided targeted therapy, PI3KCA mutations do not appear to be involved in the resistance mechanism of anti-HER2 therapies.

Once the oncogenic mutation is acquired, it can become a driver excluding generally other oncogenic mutations. It can then impact the tumor microenvironment and grow by promoting tumor angiogenesis. This is what we observe in our study. Indeed, tumors with a PI3KCA mutation are larger and trend to be associated with more tumor invasion of microvessels. This probably makes the tumors more aggressive. Similarly, the increase rate of PI3KCA mutations in the women who underwent several hepactectomies for tumor relapse even when liver resections were complete is in favor of the emergence of the clones carrying resistance mutations.

The persistence of PI3KCA mutations over time and despite one or two R0 hepatectomies, as well as the delay between 2 hepatectomies, mainly signify the presence of a reservoir of tumor cells in the form of dormant cells or stem cells [22]. These cells would preferentially carry the PI3K mutations conferring on them this property. They can also circulate in the bloodstream and be identified as prognostic and/or predictive markers to guide therapeutic decisions.

These findings raise more questions than answers. Common knowledge dictates that cancer progresses in steps [23, 24] and treatment strategies have been directed at the last step of “evolution”. The basis of systemic palliative treatment has been based on trial and error and our findings raise the question whether a better strategy might be to base our strategies on tumor lineage. In other words, tumor cells might have a common origin but might have taken different evolutionary paths during time. A proposition could be to surgically remove and analyze all tumors to identify inter tumor “lineage” variation which in turn would lead to multi-lineage treatment strategies with the ultimate goal to eradicate all malignant cells.

Dan Frumkin et al., developed a way of reconstructing cell lineage trees from genomic variability caused by somatic mutations. This method was applied to cancer and for the first time a lineage tree of neoplastic and adjacent normal cells was reconstructed [25]. Other studies have also come to similar findings when examining chemotherapies effect using radiolabeling and imaging studies. Both methods support the idea of multi-lineage and genetic heterogeneity of metastases disease [24, 26].

This relatively small series study has some inherit limitation. There is no statistical power to make a definitive statement about clinical significance of our findings however these findings have not been presented before and can become the basis for further studies on the tumor heterogeneity and changes overtime. As a specialized center, the primary tumor tissue is usually not available for genetic studies which is important to complete the picture and lineage link. Furthermore, women with breast cancer liver metastases, usually present years after primary tumor discovery which makes it challenging to obtain primary tumor tissue and perform these genetic mutation detections.

## Conclusion

In conclusion, PIK3CA mutations are frequently observed in breast cancer liver metastases and persist over time. The frequently observed mutations in PIK3CA that have been associated with resistance to chemotherapy, anti-HER2 or anti-estrogen therapies could be involved in the recurrence of liver metastases and might be a target in the future[27]. The identification of circulating tumor cells carrying PI3KCA should become a useful biomarker in the routine practice of breast cancer to prevent tumor recurrence but also to overcome the problems of intra- and inter-tumoral heterogeneity, as well as temporal, of the current biomarkers, which are hormonal receptor or HER2 expression measurements whose evolutions are not re-evaluated during treatments or in all metastatic sites.

## Abbreviations

BCLM: Breast Cancer Liver Metastases
HER2: Human epidermal growth factor receptor
PIK3CA: Phosphatidylinositol-4,5-Bisphosphate 3-Kinase Catalytic Subunit Alpha
